# SCB-YOLOv5: a lightweight intelligent detection model for athletes’ normative movements

**DOI:** 10.1038/s41598-024-59218-w

**Published:** 2024-04-14

**Authors:** Qing Du, Lian Tang, Ya Li

**Affiliations:** 1 School of Resource Environment and Safety Engineering, University of South China, Hengyang, 421001 China; 2 School of Sports Science and Engineering, Hunan Institute of Engineering, Xiangtan, 411100 China; 3 School of Electrical Information Engineering, Hunan Institute of Engineering, Xiangtan, 411100 China

**Keywords:** Digital sports, Action recognition, Deep learning, Attention module, Engineering, Mathematics and computing

## Abstract

Intelligent detection of athlete behavior is beneficial for guiding sports instruction. Existing mature target detection algorithms provide significant support for this task. However, large-scale target detection algorithms often encounter more challenges in practical application scenarios. We propose SCB-YOLOv5, to detect standardized movements of gymnasts. First, the movements of aerobics athletes were captured, labeled using the labelImg software, and utilized to establish the athlete normative behavior dataset, which was then enhanced by the dataset augmentation using Mosaic9. Then, we improved the YOLOv5 by (1) incorporating the structures of ShuffleNet V2 and convolutional block attention module to reconstruct the Backbone, effectively reducing the parameter size while maintaining network feature extraction capability; (2) adding a weighted bidirectional feature pyramid network into the multiscale feature fusion, to acquire precise channel and positional information through the global receptive field of feature maps. Finally, SCB-YOLOv5 was lighter by 56.9% than YOLOv5. The detection precision is 93.7%, with a recall of 99% and mAP value of 94.23%. This represents a 3.53% improvement compared to the original algorithm. Extensive experiments have verified that our method. SCB-YOLOv5 can meet the requirements for on-site athlete action detection. Our code and models are available at https://github.com/qingDu1/SCB-YOLOv5.

## Introduction

The movements of aerobic gymnasts must be standardized, as this directly impacts their safety. Scientific and standardized movements can reduce or even eliminate the risk of injury. The new development stage of digital sports embodies the deep integration and interaction of “digital” and “sports”^1^. Detecting and analyzing the behavior of aerobic athletes can help promote innovation in sports education, including actively promoting sports image recognition^2^, 3D motion modeling analysis^3^, and live streaming^4^.

In recent years, human action recognition based on deep learning^5–8^ has found extensive applications in smart cities, industrial production, intelligent transportation systems, and other fields. The analysis of automated video content holds the potential to significantly advance monitoring capabilities, encompassing action recognition, target tracking, and pedestrian re-identification. Such advancements offer a practical approach to recognizing athletes’ actions.

Precisely categorization and assessment of athlete behavior through visual data involves leveraging computer vision technology to predict action categories and evaluate action quality. Existing object detectors serve as important references for recognizing athlete behavior. However, deep neural models have a large number of model parameters, and their calculations are complex, imposing high demands on hardware computing capabilities, memory bandwidth, and data storage. This makes it costly to use them for practical sports education. Given the specialized and standardized nature of athletes’ movements, further research is needed on existing recognition methods.

**Figure 1:**
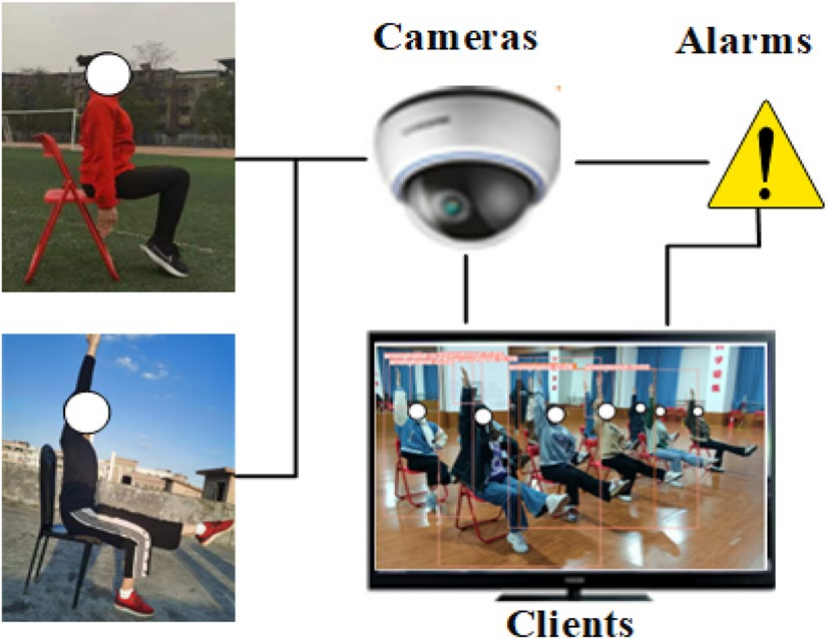
Our intelligent detection system for athlete normative behavior, including RGB cameras, clients, and alarms.

In summary, we have proposed a lightweight intelligent detection model: SCB-YOLOv5. This model is capable of analyzing human behavior in video images, identifying various types of actions, and responding promptly to specific circumstances, as illustrated Fig. 1.

We summarize the main contributions are as follows:

- We collect images of aerobic athletes, classify their actions anew, and establish the ANBD dataset for recognizing their behavior.
- In terms of model optimization, the SCB-YOLOv5 model is proposed. It features a more lightweight backbone and incorporates a weighted BiFPN to enhance the performance of the original model.
- We compared and analyzed comprehensive experiments with several approaches. The quality metrics of the target detectors were evaluated to validate the effectiveness of our approach.

## Related works

### Target detection algorithms

**Figure 2:**
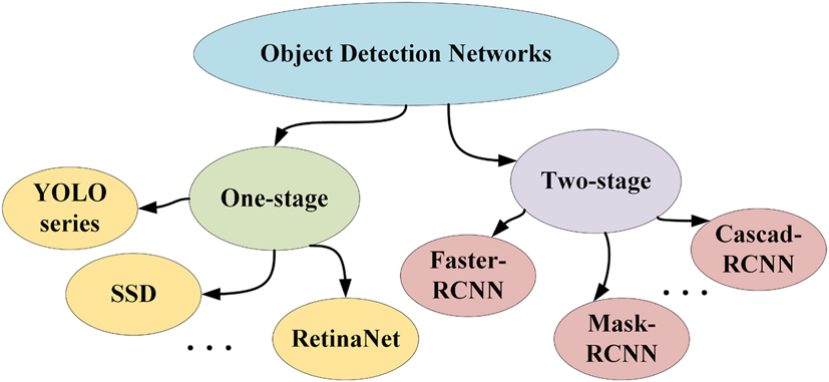
Existing target detection algorithms based on deep learning, where one-stage include YOLO series^13–17^, SSD^18^, RetinaNet^19^, etc. and two-stage networks include Faster-RCNN^20^, Mask-RCNN^21^, Cascad-RCNN^22^, etc.

Target detection algorithms rely on convolutional operations^9^. Based on their framework structure, these algorithms can be categorized into two types: one-stage and two-stage models, as depicted in Fig. 2. One-stage algorithms generally prioritize high real-time performance and simplicity, often at the expense of detection accuracy. Conversely, two-stage algorithms utilize the regional proposal network (RPN) to generate suggestions and then employ a fully connected layer to produce category predictions and bounding boxes, resulting in higher detection accuracy. Despite having fewer network layers, some one-stage algorithms^10–12^ have recently outperformed two-stage networks in both accuracy and speed, and are widely employed in automated detection applications.

### Actions recognition

Deep learning-based algorithms for recognizing athletes’ normative actions, encompassing classification and detection. These capture video sequence images of athletes’ movements and employ convolutional neural networks for model training to predict their actions. Numerous scholars have delved into human action recognition from a kinesiology perspective.

Zhang et al.^23^ proposed a method that relies on multimodal sequence fitting to detect the behavior of college basketball players. This method combines motion patterns and visual motion features captured by cameras. Integrating global and local motion patterns can significantly improve the performance of group behavior recognition. Julian Fritsch et al.^24^ introduced an intelligent detection algorithm for recognizing the post-scoring emotions of volleyball players, achieving a precision rate of 80.09%. Zhao et al.^25^ focused on deep video analysis, extracting frame sequences as inputs for a 3D convolution-based deep neural network. This algorithm automatically captures spatio-temporal features of athlete behavior, thereby enhancing the accuracy of recognizing body movements.

### Our dataset

Deep learning-based target detectors necessitate a substantial number of pre-labeled samples to enhance accuracy and generalization capability^26^. We categorized movement classification based on fundamental body postures, basic techniques, and coordination. To create the athlete normative behaviors dataset (ANBD), we utilized pictures and video footage captured by members of a university aerobics team in Hunan Province, China. These videos were edited to extract one frame every 5 s, resulting in a collection of 2121 images showcasing various athletes in different scenes and angles.

**Figure 3:**
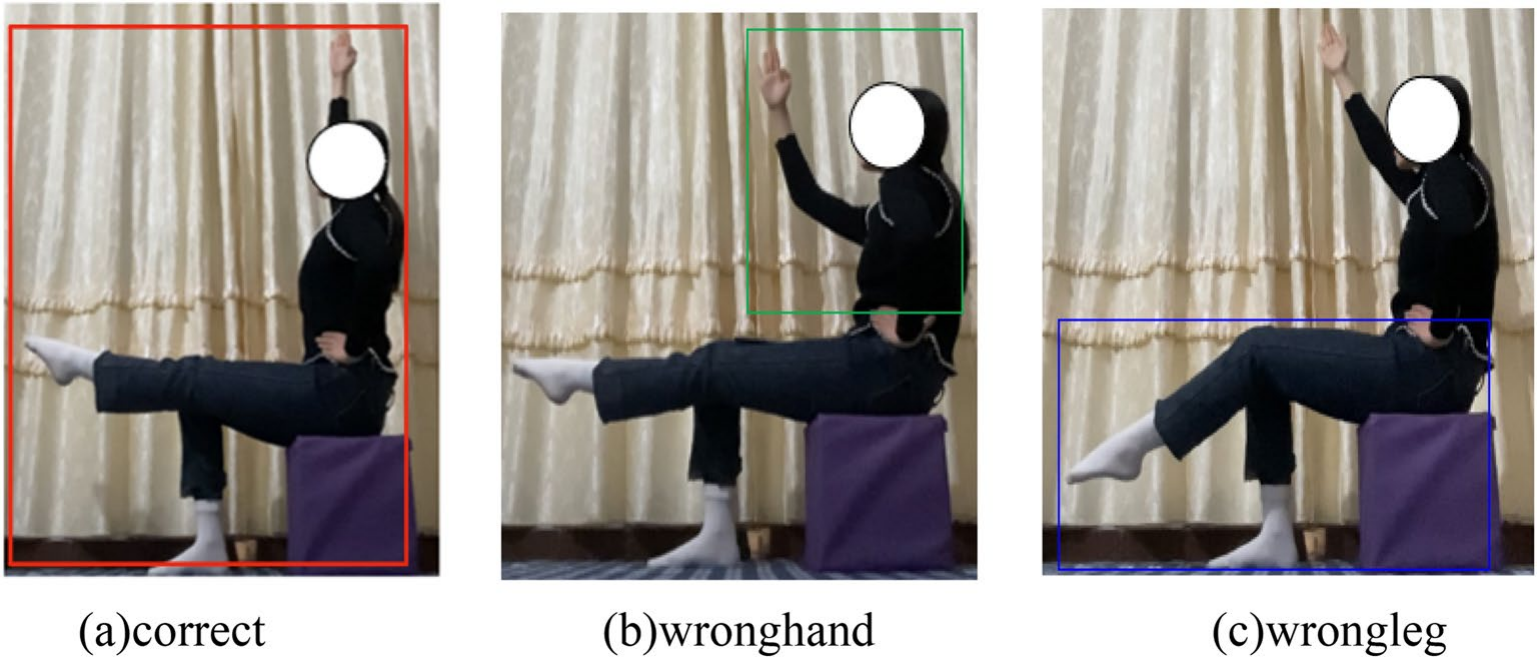
Classifying the movement behaviors of aerobics players, we categorized the common movements of the participants as follows: (**a**) the red box in the middle indicates that the athlete’s arms and legs are straight, demonstrating standard movements; (**b**) the green box in the middle indicates that the athlete’s hands are bent at the elbows, which is an incorrect movement that does not meet the normative requirements; and (**c**) the blue box in the middle indicates that the athlete’s calves are bent, which is an incorrect movement that does not meet the standard movements.

The determination of whether a movement constitutes a standardized action relies on the varying amplitudes of the athlete’s arm, elbow, leg, and other movements, as illustrated in Fig. 3. Among these, (a) depicts the standardized correct action, labeled “correct”; (b) illustrates the wrong hand action, labeled “wronghand”; (c) portays the wrong leg action, labeled “wrongleg”.

## Method

### SCB-YOLOv5 model

All the images used in this research were obtained from the Hunan Institute of Engineering in Xiangtan, China, with 216 individuals, comprising 19 teachers and 197 students. All volunteers who participated in the photo shoots were informed about the data usage and provided consent for the research presented in this paper.

**Figure 4:**
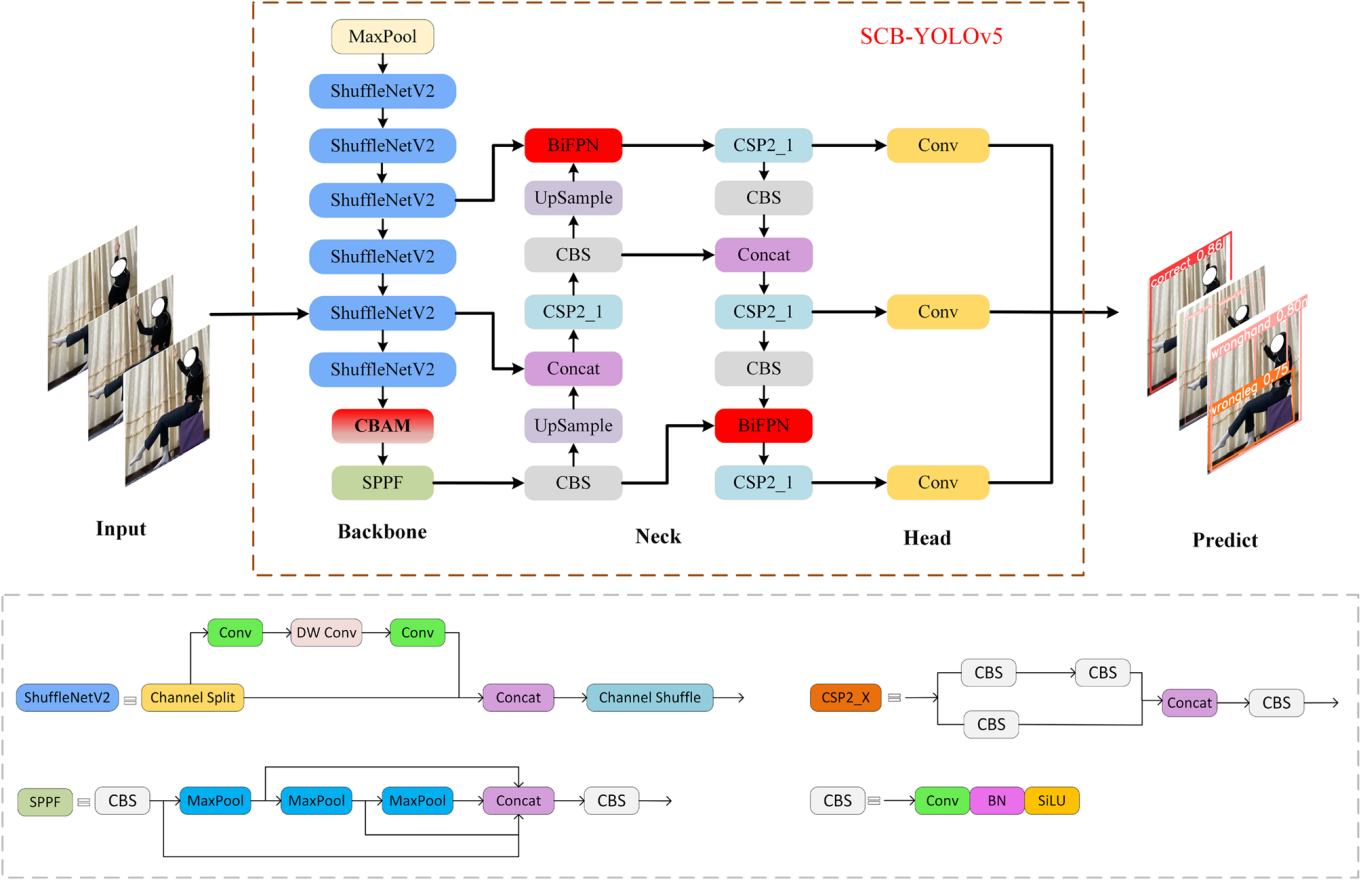
In the SCB-YOLOv5 structure, we primarily updated the backbone network and the Neck part of the YOLOv5.

The overall structure of the SCB-YOLOv5 is derived from YOLOv5 and mainly consists of five components: input, Backbone, Neck, Head, and Predict. The specific structure is shown in Fig. 4. ShuffleNet v2^27^ servers as the backbone to achieve a more lightweight design, integrating the CBAM at the base layer to capture additional feature information. The Neck component comprises BiFPN, which integrates semantic information from the deep network into the shallow network. Finally, the output predicts image features and generates the bounding box with the highest confidence based on the size of the target.

### Mosaic-9

**Figure 5:**
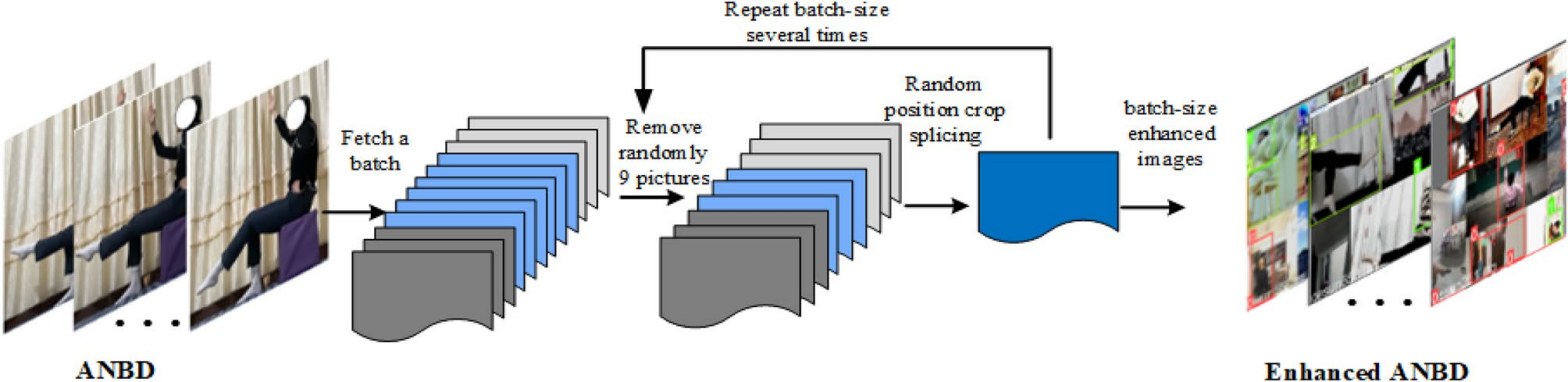
Mosaic-9. Using this strategy can effectively increase the amount of data, and our experiments verify that it has a positive effect on the subsequent model training.

The mosaic data enhancement in Yolov4^14^ randomly selects four images from the training set and combines their contents to create synthesized images directly used for training. This data augmentation method could enhance the ability of YOLOv4 to recognize objects in complex backgrounds. Therefore, we employ the Mosaic-9^17^ enhancement in YOLOv5, as illustrated in Fig. 5. Initially, a batch of images is randomly selected from the dataset, followed by the random selection of nine images from the extracted set. These pieces are then cut and stitched together to create a new image. This process is repeated batch size times (batch size refers to the number of images extracted from the dataset), resulting in an enhanced image of the specified batch size.

### ShuffletNet V2

ShuffleNet V2^27^ introduces new enhancements to the structure of ShuffleNet V1^28^. The ShuffleNet V2 network structure is illustrated in Table 1. A 1 × 1 convolutional layer is incorporated to blend the features before the global average pooling. Efficient utilization of each stage enables an increase in feature channels and enhances network capacity. Notably, half of the feature channels in each block are directly transmitted to the subsequent one. This mechanism resembles feature reuse, akin to the concepts of DenseNet^29^ and CondenseNet^30^. Such a structure enables information communication between different channel groups and enhances reliability.

**Table 1:** ShuffleNet V2 structure, for each stage, its first block is required to be doubled, and the step size strips are all equal to 2.

Layer	Out size	KSize	Stride	Repeat	Output channels			
					0.5 ×	1 ×	1.5 ×	2 ×
Image	224 × 224				3	3	3	3
Conv1 MaxPool	114 × 114 56 × 56	3 × 3 3 × 3	2 2	1	24	24	24	24
Stage2	28 × 28 28 × 28		2 1	1 3	48	116	176	244
Stage3	14 × 14 14 × 14		2 1	1 7	96	232	352	488
Stage4	7 × 7 7 × 7			1 3	192	464	704	976
Conv5	7 × 7	1 × 1	1	1	1024	1024	1024	2048
GlobalPool	1 × 1	7 × 7						

### Convolutional block attention module

**Figure 6:**
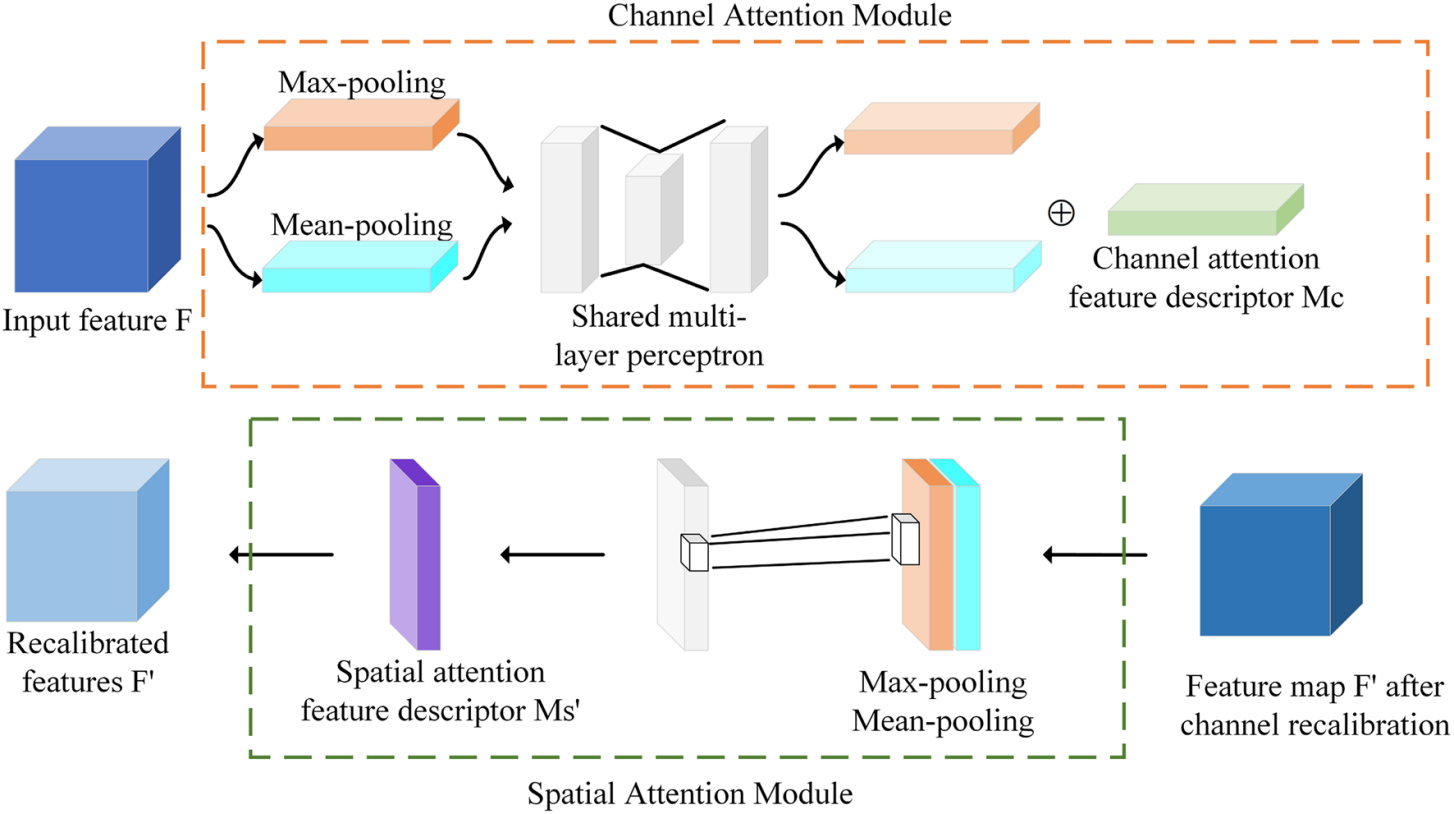
Structure of CBAM.

Replacing the YOLOv5 backbone with the lightweight ShuffleNet V2^27^ results in the loss of certain image feature information. To preserve more high-level semantic information, CBAM^18^ is added after the ShuffleNet V2, thereby directing more attention towards the significant aspects of the image. The CBAM attention mechanism is illustrated in Fig. 6.

CBAM comprises a channel attention module and a spatial attention module. It can effectively prioritize information crucial to the current task goal, enhancing the relevance of the extracted features from convolutional layers, capturing more comprehensive high-level semantic information, and improving target recognition. The calculation formula is as follows:

1 $${F}{\prime}={M}_{C}(F)\otimes F$$

2 $${F}^{{\prime}{\prime}}={M}_{S}({F}{\prime})\otimes {F}{\prime}$$

while, $$F\in {R}^{C*H*W}$$ is the input feature, $${M}_{C}\in {R}^{C*1*1}$$ is the one-dimensional convolution of the channel attention module, $${M}_{S}\in {R}^{1*H*W}$$ is the spatial attention module, $${F}^{\mathrm{^{\prime}}}$$ is the output feature after passing through the channel attention module, and $${F}^{\mathrm{^{\prime}}\mathrm{^{\prime}}}$$ is the final output feature.

### Fusing the neck part of the BiFPN

**Fig.7:**
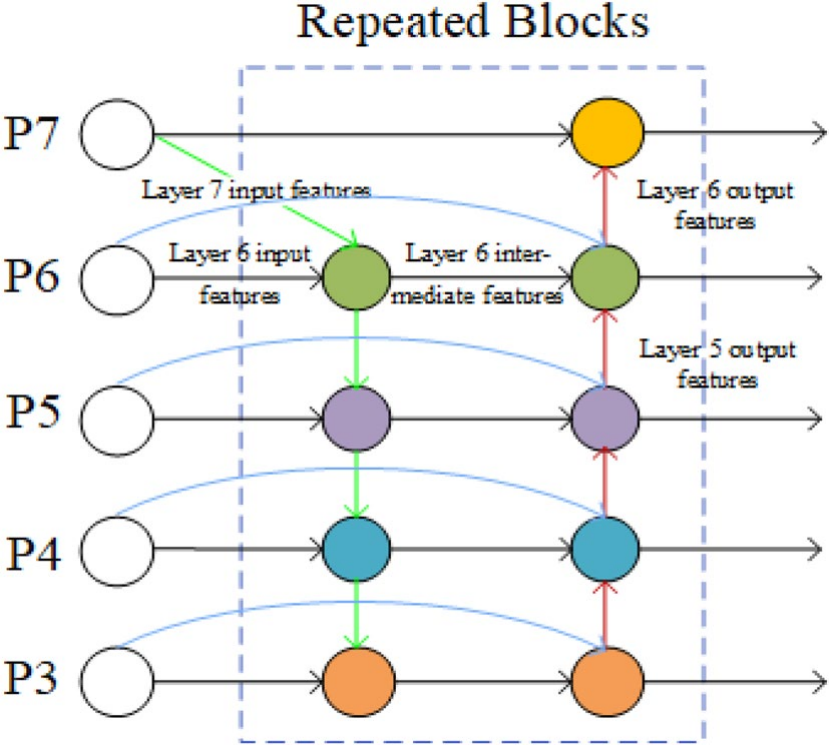
BiFPN Node Diagram. The purple curve connects input nodes and output nodes within the same layer. The blue curve conveys semantic information of high-level features, while the red curve conveys positional information of bottom-level features.

The significance of analyzing images at multiple scales arises from the inherent complexity of images. Real-world scene images encompass a multitude of large and small target objects, each bearing diverse information such as size, position, color, and other attributes. Hence, relying solely on the bottom-up FPN pyramid structure has the potential to overlook information across different scales. To address this issue, Mingxing Tan et al.^10^ proposed BiFPN, a straightforward and efficient feature pyramid network, as depicted in Fig. 7.

The multi-scale feature fusion of BiFPN aims to aggregate features with different resolutions. Because the input features have different resolutions, BiFPN uses a band-weighted feature fusion method (Fast Normalized Fusion).

3 $$O={\sum }_{i}\frac{{w}_{i}}{\in +{\sum }_{j}{w}_{j}}\cdot {I}_{i}$$

where $\in$ = 0.0001 is used to avoid numerical instability, and $w$ is a learned parameter, similar to an attention mechanism, used to distinguish the significance of various features in the feature fusion process.

## Experimental results and discussion

### Experiment settings

All our experiments were conducted using the Windows 10 system, utolizing the PyTorch deep learning framework. The processor employed is Intel(R) Core(TM) i5-10400F CPU @ 2.90 GHz, with 16 GB of RAM, and the GPU model is NVIDIA GeForce RTX 1650 graphics card with 4 GB of memory.

The dataset utilized for the experiments is ANBD, which consists of 2121 images as detailed in our dataset. The dataset is divided into training and validation sets in an 8:2 ratio. Model training encompasses 100 epochs, with a batch size set to 2, and the initial learning rate set to 0.01.

### Model performance evaluation metrics

Average precision (AP) and mean average precision (mAP) are commonly used in target detection to evaluate the detection algorithms. The calculation formulas are presented in Eqs. (4) and (5):

4 $$AP={\int }_{0}^{1}PdR$$

5 $$mAP=\frac{\sum_{i=1}^{k}A{P}_{i}}{k}$$

where AP is the average precision of a single category, mAP is the mean of the AP values of all categories, F1 is the reconciled mean of P and R, P is the precision rate, R is the recall rate, and k is the number of detected categories.

**Figure 8:**
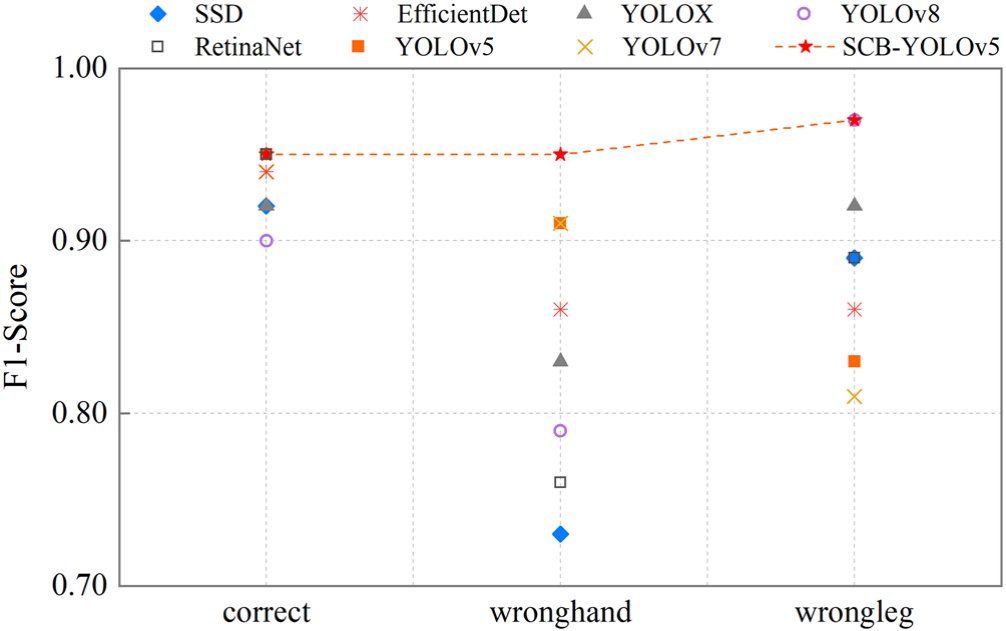
The F1-SCORE of detectors, where SCB-YOLOv5 has the highest F1-SCORE, while the other detectors exhibit less favorable detection results for the classification of wronghand, again confirming Table 2 above.

AP is a common metric for evaluating the overall performance of a detector. However, excessive emphasis on labeled positive samples while pursuing AP can result in a high number of false detections. In practical evaluations, the F1-Score is employed as the evaluation criterion, offering a more balanced and effective measure of overall performance. Following 100 epochs of training, a relatively high confidence threshold (confidence = 0.5) is usually set to filter out a large number of false detection frames. Subsequently, the performance is analyzed using the F1-SCORE. As shown in Fig. 8.

### Comparison with detectors results

Extensive experiments were conducted, including multi-group visual quality comparisons and image quality assessment, as shown in Table 1. (1) Among the original models, we found the YOLOv8 delivers the best detection performance. However, it comes with a significantly larger number of model parameters compared to YOLOv5, albeit offering a comparable detection performance at a slightly highter cost. Therefore, the experiment aims to enhance YOLOv5. (2) SCB-YOLOv5 greatly reduces the network parameters and minimizes hardware computation. The mAP reaches 94.23%, which is 3.53% higher than YOLOv5.

### Detection demos

**Figure 9:**
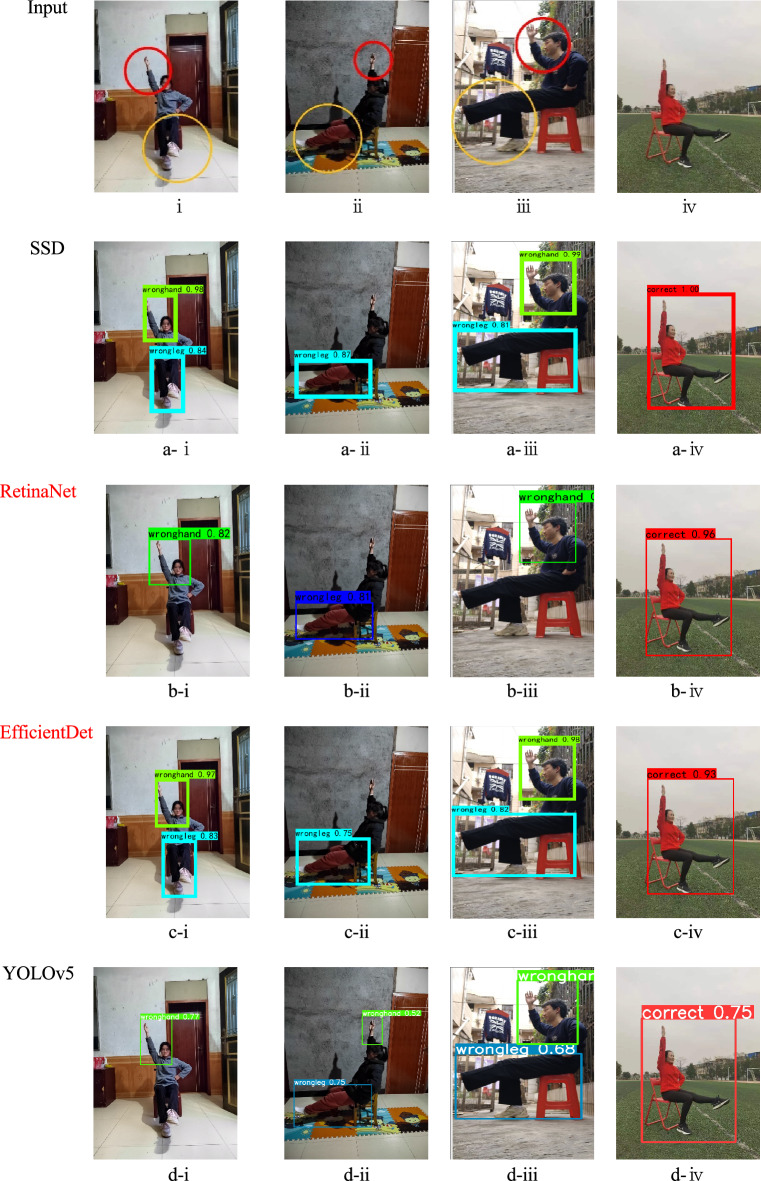

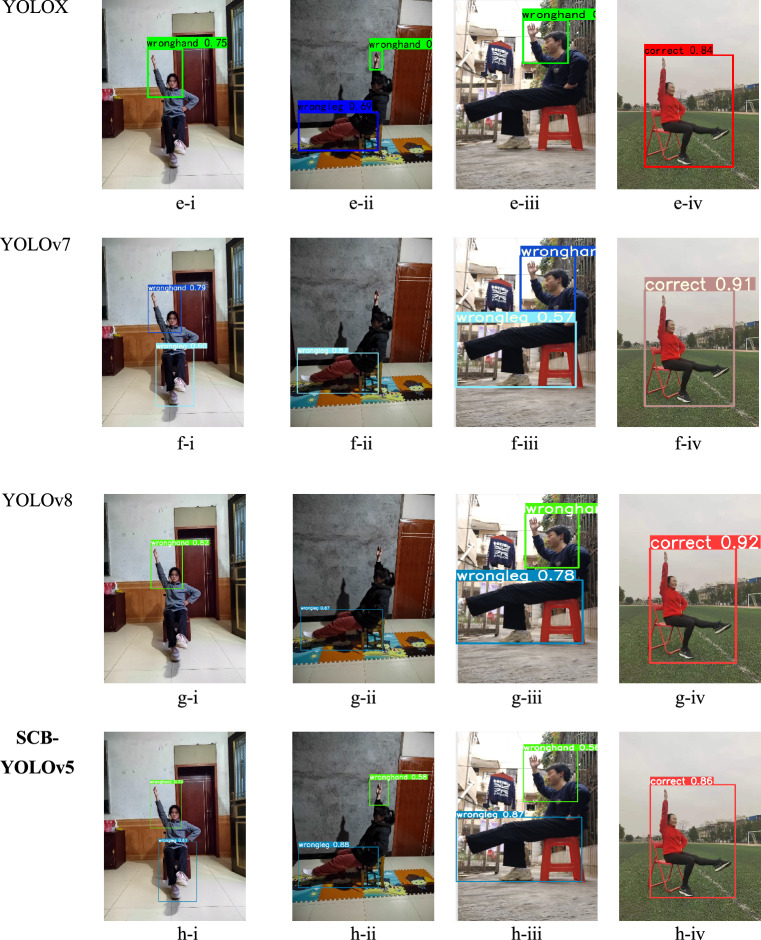
Detection demos with SSD, YOLOv5, YOLOX, YOLOv7, and YOLOv8 are shown in the figures i-iv depict diagrams of athlete behavior in various scenarios. Hand irregularities are marked in the red circles, foot irregularities in the yellow circles, and iv showcases correct behavior demonstrations. In figures a-i to f-iv, each detection box displays the detection confidence level, where higher values indicate greater confidence in the results of that detection.

The detection demos are shown in Fig. 9, corresponding to the data in Table 2. In the SSD algorithm figures a-ii, b-ii, c-ii, f-ii, and g-ii miss the detection of “wronghand” behavior. Figure a-iv has the highest sensitivity with a detection confidence of 100%. Conversely, the YOLOX and RetinaNet algorithms are less effective in detecting the “wrongleg” behavior. Figures b-iii and e-iii demonstrate the mission of “wrongleg”. Finally, SCB-YOLOv5 achieves accurate detection results.

**Table 2:** Comparison of the detector results.

Model	Backbone	Params (M)	AP			Recall	mAP
			correct	wronghand	wrongleg		
SSD	VGG		**97.89**	79.44	*93.62*	86.77	90.32
RetinaNet	ResNet50	34.0	95.90	81.53	92.03	81.06	89.82
EfficientDet	Efficientnet	*6.6*	91.64	80.83	82.30	89.18	84.92
YOLOv5	CSPDarknet53	7.2	96.15	84.04	91.92	89.16	90.70
YOLOX	Darknet-53	9.0	96.10	*89.92*	84.98	89.16	90.33
YOLOv7	E-ELAN	37.2	96.80	81.50	90.80	**99.00**	89.70
YOLOv8	CSPDarknet53	11.2	*97.09*	84.53	93.18	*98.00*	*91.56*
**SCB-YOLOv5**	ShufleNet-v2	**3.1**	95.20	**93.00**	**94.50**	**99.00**	**94.23**

The best experimental results are labeled in bold, and the second-best results are indicated in italic.

**Figure 10:**
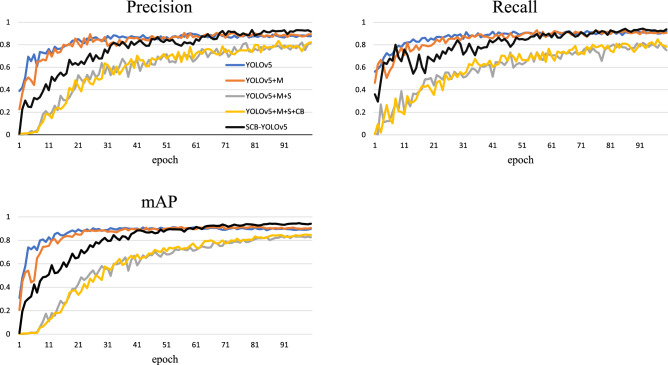
Line graph comparing the performance of each improvement experiment, where the horizontal coordinate is the 100epoch of the training of each algorithm model and the vertical coordinate is the index value.

In developing the SCB-YOLOv5, we meticulously documented the impact of each adjustment in the experiment, with a specific focus on the changes in Precision, Recall, and mAP. As shown in Fig. 10, the black line graph demonstrates that SCB-YOLOv5 outperforms the other methods in each performance metric after training stabilization.

### Ablation study

To evaluate the effectiveness of the improved module on the SCB-YOLOv5, the ablation study was performed on our dataset. The results are shown in Table 3. After replacing the original backbone with the ShuffleNet V2 network, there was a decrease due to the reduced complexity of the model. This was done to maintain the detection performance of the model. Consider incorporating an attention mechanism after the ShuffleNet V2 backbone, and subsequently integrating BiFPN across multi-scale features to improve SCB-YOLOv5. Through extensive experiments, it has been proven that the adopted optimization strategy can enhance the accuracy of detecting. The mAP value has increased by 3.53 percentage points compared to the original.

**Table 3:** Results of ablation experiments.

YOLOv5	Mosaic-9	ShufleNet-v2	CBAM	BiFPN	mAP
√					90.70
√	√				90.75
√	√	√			83.33
√	√	√	√		85.59
√	√	√	√	√	94.23

## Conclusion

In this study, we introduce a dataset for detecting the actions of aerobic athletes. A lightweight algorithm SCB-YOLOv5 is designed to recognize and regulate actions. To innovate the application of digital sports teaching processes.

The results of multiple sets of experiments show that the enhanced model has a more significant impact on recognizing athletes’ irregular hand and leg movements, outperforming other detectors. This finding holds major significance in promoting the sustainable and healthy development of “Internet + Education”.

## Data Availability

Datasets generated and/or analyzed during the current study are available from the corresponding author upon reasonable request.

## Code availability

Our code and models are available at https://github.com/qingDu1/SCB-YOLOv5. Our model code is open data. However, the data set used in this article is not available, and the authors can be contacted if necessary.
